# Clinical Applications of Somatostatin Receptor (Agonist) PET Tracers beyond Neuroendocrine Tumors

**DOI:** 10.3390/diagnostics12020528

**Published:** 2022-02-18

**Authors:** Rasmus Helgebostad, Mona-Elisabeth Revheim, Kjersti Johnsrud, Kristine Amlie, Abass Alavi, James Patrick Connelly

**Affiliations:** 1 Division of Radiology and Nuclear Medicine, Oslo University Hospital, P.O. Box 4956 Nydalen, 0424 Oslo, Norway; helgebostad@gmail.com (R.H.); monar@ous-hf.no (M.-E.R.); kjohnsru@ous-hf.no (K.J.); kkraml@ous-hf.no (K.A.); 2 Institute of Clinical Medicine, University of Oslo, P.O. Box 1171 Blindern, 0318 Oslo, Norway; 3 Department of Radiology, Hospital of the University of Pennsylvania, 3400 Spruce Street, Philadelphia, PA 19104, USA; abass.alavi@uphs.upenn.edu

**Keywords:** somatostatin, PET, imaging, review, inflammation, benign, malignant, sarcoidosis, atherosclerosis, osteomalacia

## Abstract

Somatostatin receptor (SSTR) agonist tracers used in nuclear medicine scans are classically used for neuroendocrine tumor diagnosis and staging. SSTR are however, expressed more widely in a variety of cells as seen in the distribution of physiological tracer uptake during whole body scans. This provides opportunities for using these tracers for applications other than NETs and meningiomas. In this qualitative systematic review, novel diagnostics in SSTR-PET imaging are reviewed. A total of 70 studies comprised of 543 patients were qualitatively reviewed. Sarcoidosis, atherosclerosis and phosphaturic mesenchymal tumors represent the most studied applications currently with promising results. Other applications remain in progress where there are many case reports but a relative dearth of cohort studies. [^18^F]FDG PET provides the main comparative method in many cases but represents a well-established general PET technique that may be difficult to replace, without prospective clinical studies.

## 1. Introduction

Somatostatin is a cyclic peptide hormone with two active forms consisting of either 14 or 28 amino acids regulating specific and selective functions depending on the location. Somatostatin-producing cells are typically neurons or endocrine-like cells found in high density in the central and peripheral nervous systems, the endocrine pancreas, liver, spleen and in the gut. They can also be found in smaller numbers in the thyroid, adrenals, submandibular glands, kidneys, prostate, placenta blood vessels, and immune cells [1].

Somatostatin bears several endocrine functions including pituitary regulation of growth hormone (GH) and thyroid stimulating hormone (TSH, inhibiting GH/TSH secretion through somatostatin secretion from the hypothalamus). Moreover, it has an inhibitory effect on various gastrointestinal functions, including gastric acid secretion, gastric emptying, intestinal motility, release of insulin and glucagon and various gastrointestinal hormones [2]. The peptide binds to the G-coupled receptor SSTR, one of a large class of cellular membrane receptor proteins with seven transmembrane segments containing a peptide binding region at the external surface and an internal signaling system based on G-proteins and changes in guanosine phosphorylation. The receptor acts as a switch that is activated by binding somatostatin. SSTR is expressed by nerve cells, many neuroendocrine cells and inflammatory cells such as lymphocytes, monocytes/macrophages, peripheral blood mononuclear cells and thymocytes [3]. There are five receptor subtypes SSTR1-5 expressed in different ratios in different organ systems. In the peripheral blood mononuclear cells and in the spleen, mainly SSTR subtypes 2 and 3 are found; in the thymus, mainly SSTR subtypes 1, 2, and 3; in macrophages and dendritic cells, mainly SSTR subtype 2; in B lymphocytes, mainly SSTR subtype 3; and in T lymphocytes, SSTR subtypes 1 through 5 [4,5,6,7]. Receptor distribution is shown in Table 1.

**Figure 1:**
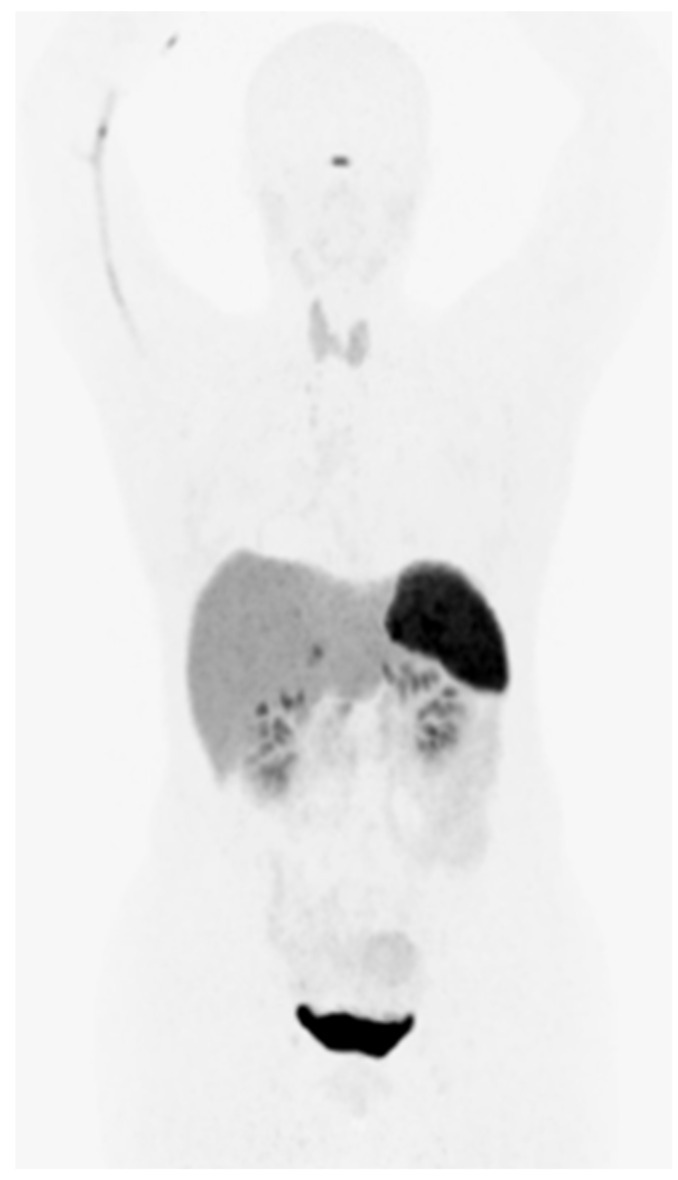
Physiological uptake of [68Ga]Ga-DOTATOC. An imaging protocol based on EANM guidelines was used [9]. A 145MBq bolus of [68Ga]Ga-DOTATOC was injected and imaging performed after 60 min.

The SST receptor can be targeted with synthetic somatostatin analogues exhibiting both agonist and antagonist activity. Antagonist based tracers are not yet well established and are not included in this review. Octreotide was the first somatostatin agonist analogue developed for clinical application and is currently used in the treatment of neuroendocrine tumors. PET (positron emission tomography) tracers are created by binding octreotide (or analogue) with a metal radionuclide using a chelating agent such as DOTA (1,4,7,10-tetraazacyclododecane-1,4,7,10-tetraacetic acid). This was achieved successfully with gallium-68 ([^68^Ga]) and copper-64 ([^64^Cu]) for PET use [5] in combination with DOTA-D-Phe-Tyr3-octreotide (DOTATOC), DOTA-1-NaI(3)-octreotide (DOTANOC), or DOTA-D-Phe-Tyr3-octreotate (DOTATATE), which differ mainly in their affinity to the various receptor subtypes (SSTR1-5). DOTATOC is more selective to type 2 and 5, DOTANOC 2,3 and 5, and DOTATATE type 2 [8]. Physiological uptake of [^68^Ga]Ga-DOTATOC is shown in Figure 1.

Somatostatin receptors (SSTR) tracers are routinely used in diagnostics and therapy of neuroendocrine tumors (NET) [5], particularly gastroenteropancreatic NETs. Meningiomas are non-neuroendocrine tumors that frequently exhibit a high level of SSTR 2 expression and are not infrequent incidental findings on SSTR-PET scans performed for NET diagnosis. As a result, literature continues to accumulate regarding the use of SSTR PET for meningioma diagnosis and treatment with this provides a good example of a non-NET application. There are already a number of reviews detailing this application, thus meningiomas are excluded from this review [10,11].

SSTRs are expressed in a multitude of cells around the body that could suggest other potential uses in PET imaging. In this review, we consider these commonly available SSTR-PET tracers in diagnostic uses other than NETs and meningiomas and present an overview of reported cases and applications.

## 2. Materials and Methods

A search was performed in PubMed (1993–February 2021) for the most common PET tracers in clinical use using keywords DOTATOC, DOTATATE and DOTANOC. Neuroendocrine tumors were excluded using the “NOT” command for keywords “neuroendocrine” and “NET”. Pheochromocytoma and paraganglioma were considered as part of the neuroendocrine tumor group and excluded. As mentioned in the introduction, studies involving meningiomas were also excluded.

**Figure 2:**
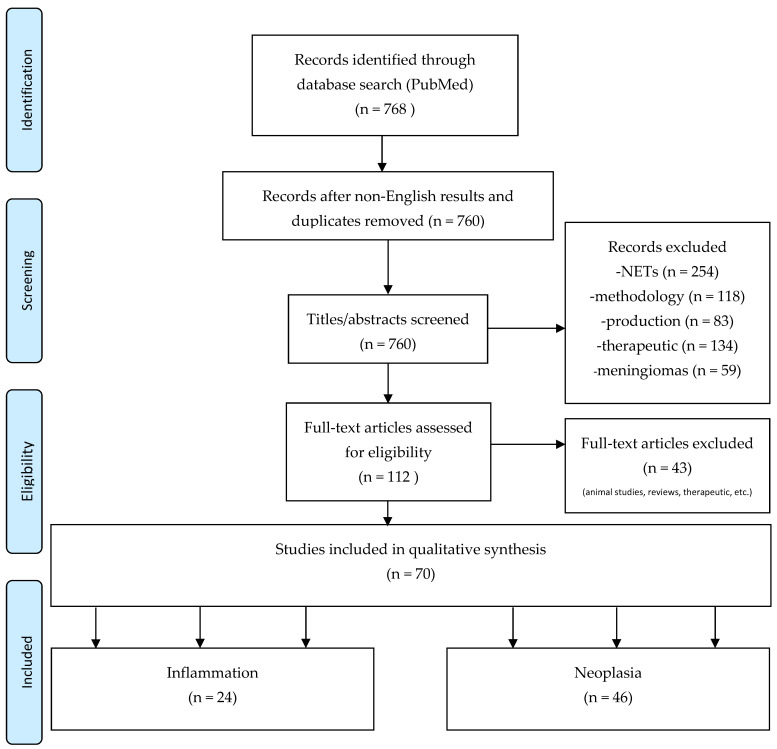
Flow chart of the review process based on PRISMA methodology [12].

For this review we included observational cohort studies and case reports. Patients with indications other than NETs and meningiomas who had diagnostic radiolabeled somatostatin receptor analogue PET imaging, using DOTANOC, DOTATOC, DOTATATE tracers were included. Publications with emphasis on technical aspects related to generators, radiochemistry, animal models, experimental reports or physics were excluded. A flow diagram of the search process is shown in Figure 2.

## 3. Results

Pubmed literature search yielded 768 results. The exclusion of non-English results and duplicates resulted in 760 articles. The titles/abstracts revealed 254 articles concerning NETs, 59 articles on meningiomas, 118 general articles about SSTR PET/CT without specific diagnostic focus, 83 articles regarding production and 134 involving therapeutic use. These articles were all excluded. A total of 112 full-text articles were assessed for eligibility. Using the same criteria, 43 full-text articles were excluded and 70 studies with a total of 543 patients were included in a qualitative systematic review. A PRISMA flow diagram [9] of the process is shown in Figure 2.

The literature can be grouped into two broad sections on inflammation including cardiovascular disease and ischemia, and neoplasia divided into benign and malignant tumors. An overview of the literature and references is given in Table 2. As can be expected for novel or incidental applications of SSTR tracers, the literature consists of relatively small cohort studies and a number of case reports. Table 3 and Table 4 list details of cohort studies for each subject area. The case studies listed in Table 2 are reviewed in the Section 4.

SSTR tracer imaging protocols described in each study largely conform to standard protocols with regard to timing and activity used for neuroendocrine tumors [9]. Typically, a tracer activity of 75–300 MBq was administered and imaging performed 1 h (±30 min) after injection. Two exceptions are the study on disease activity in sarcoidosis by Sharma et al. [13] with activities of 1110–1480 MBq and the tumor-induced osteomalacia study by Breer et al. [14] with imaging performed 20 min after injection.

**Table 2:** Overview of pathology (other than NET and meningioma) yielded by the literature search.

**Inflammatory Disease**	**Cohort Studies** **(Table 3)**	**Case Reports**
Takayasu arteritis		1 ([15])
Graves ophthalmopathy		2 ([16,17])
IgG mediated lymphadenopathy		1 ([18])
Endometriosis	1 ([19])	
Idiopathic pulmonary fibrosis	1 ([20])	
Pulmonary tuberculosis	1 ([21])	
Sarcoidosis		
● Thoracic	2 ([13,22])	
● Cardiac	4 ([23,24,25,26])	4 ([27,28,29,30])
● Neural		1 ([31])
Myocardial infarction associated inflammation	2 ([32,33])	
Atherosclerosis		
● Coronary and carotid	1 ([34])	
● Aortic and carotid	2 ([35,36])	
Ischemia in postoperative stroke		1 ([37])
**Neoplasia**	**Cohort Studies** **(Table 4)**	**Case Reports**
*Benign*		
Phosphaturic mesenchymal tumors	13 ([14,38,39,40,41,42,43,44,45,46,47,48,49])	17 ([50,51,52,53,54,55,56,57,58,59,60,61,62,63,64,65,66])
Juvenile angiofibroma	1 ([67])	
Enchondroma		1 ([68])
Vertebral hemangioma		1 ([69])
Uterine leiomyoma		1 ([70])
*Malignant*		
Multiple myeloma	1 ([71])	1 ([72])
Epithelioid hemangioendothelioma		2 ([73,74])
Renal clear cell carcinoma		3 ([75,76,77])
Gastrointestinal stromal tumor		1 ([78])
Hepatocellular carcinoma		1 ([79])
Choroidal melanoma		1 ([80])
Osteosarcoma		1 ([81])
Oligodendroglioma		1 ([82])

**Table 3:** Summary of cohort studies relating to inflammation (abbreviations: ([^18^F]FDG, 2-deoxy-2-[^18^F]fluoro-D-glucose, Pt, number of patients).

Author	Ref	Type of Study	Pt	Radiotracer Scanner, Injected Activity and Delay before Acquisition	Comments
*Endometriosis*					
Fastrez, M. (2017)	[19]	Pilot study	12	[^68^Ga]Ga-DOTA-TATE PET/CT 2 MBq/kg, 60 min	DOTATATE showed uptake in rectovaginal deep infiltrating endometriosis and adenomyoma, but not in superficial peritoneal endometriosis or ovarian endometrioma. Immunohistochemistry used to confirm findings.
*Idiopathic pulmonary fibrosis*					
Ambrosini, V. (2010)	[20]	Prospective	14	[^68^Ga]Ga-DOTA-NOC PET/CT (GE discovery STE) 120–180 MBq, 60 min	DOTANOC uptake corresponded to areas of HRCT anomalies in patients with idiopathic pulmonary fibrosis, suggesting SSTR expression.
*Pulmonary tuberculosis*					
Naftalin, C. (2020)	[21]	Prospective	14	[^68^Ga]Ga-DOTA-NOC, PET/MR (Siemens biograph mMR) 191.7 ± 9.3 MBq, dynamic uptake image analysis on data after 60 min [^18^F]FDG	DOTANOC able to detect pulmonary tuberculosis lesions, but [^18^F]FDG was more sensitive for both active and sub-clinical lesions.
*Systemic sarcoidosis*					
Sharma, S. (2018)	[13]	Prospective	39	[^68^Ga]Ga-DOTA-NOC PET/CT (Siemens biograph mCT) 1110–1480 MBq, 30 min	27/39 patients symptomatic sarcoidosis with thoracic involvement, increased DOTANOC uptake in 25/27.
Nobashi, T. (2016)	[22]	Prospective	20	[^68^Ga]Ga-DOTA-TOC PET/CT (GE Discovery STE) 119.7 ± 29.3 MBq, 67.5 ± 8.5 min	SSTR PET positive in 19 patients, ^67^Ga-scintigraphy in 17. Suggests SSTR PET is superior in detecting sarcoidosis lesions.
*Cardiac sarcoidosis*					
Bravo, P. (2021)	[23]	Pilot study	13	[^68^Ga]Ga-DOTA-TATE, PET/CT (GE Discovery RX or Lightspeed VCT 64) 2 MBq/kg, 60 min [^18^F]FDG PET/CT	DOTATATE imaging less sensitive than [^18^F]FDG for detection of myocardial inflammation.
Gormsen, L. (2016)	[24]	Prospective	19	[^68^Ga]Ga-DOTA-NOC PET/CT(Siemens Biograph 64), 3 MBq/kg, 90 min [^18^F]FDG,	Large proportion of [^18^F]FDG-PET images were inconclusive, better diagnostic accuracy with DOTANOC in known/suspected cardiac sarcoidosis.
Pizarro, C. (2018)	[25]	Prospective	17	[^68^Ga]Ga-DOTA-TOC PET/CT(Siemens Biograph 2) 2 MBq/kg, 65 ± 28 min	Reported SSTR PET allowed for visualization of acute cardiac sarcoidosis and absent uptake correlated with use of immunosuppressants. SSTR PET may be useful in treatment response.
Lapa, C. (2016)	[26]	Prospective	15	[^68^Ga]Ga-DOTA-NOC PET/CT (Siemens Biograph 2 or mCT 64) 124 ± 31 MBq, 60 min	SSTR PET in high concordance with cardiac MRI, in patients with systemic sarcoidosis and myocardial involvement.
*Myocardial infarction associated inflammation*					
Tarkin, J. (2019)	[32]	Prospective	12	[^68^Ga]Ga-DOTA-TATE As for Tarkin (2017) [34]	DOTATATE imaging detects myocardial inflammation post infarction, both in old and ischemic injury, possibly providing a biomarker for inflammation in heart failure.
Lapa, C. (2015)	[33]	Prospective	12	[^68^Ga]Ga-DOTA-TOC PET/CT (Siemens Biograph mCT 64), 104 ± 30 MBq, 60 min	DOTATOC correlates with cardiac MRI when detecting myocardial infarction, less specific with myocarditis.
Atherosclerosis					
Tarkin, J. (2017)	[34]	Prospective	42	[^68^Ga]Ga-DOTA-TATE PET/CT (GE Discovery 690)147.8 ± 31.6 MBq, dynamic up to 90 min or static after 60 min [^18^F]FDG	DOTATATE provides quantifiable, cell-specific marker of atherosclerotic inflammation that outperforms [^18^F]FDG in coronary arteries.
Lee, R. (2018)	[35]	Retrospective	50	[^68^Ga]Ga-DOTA-TOC PET/CT(Siemens Biograph mCT 40 or 64) 185 MBq, 60 min	DOTATOC findings in thoracic aorta correlates significantly with cardiovascular risk factors.
Pedersen, S. (2015)	[36]	Prospective	10	[^64^Cu]Cu-DOTA-TATE PET/MR (Siemens Biograph mMR), 154 MBq, 85 & 299 min	DOTATATE accumulates in atherosclerotic plaques of the carotid artery. Potential for identifying vulnerable plaques.

**Table 4:** Summary of cohort studies relating to neoplasia (abbreviations: ([^18^F]FDG, 2-deoxy-2-[^18^F]fluoro-D-glucose; [^111^In]In OCT, Indium pentetreotide; Pt, number of patients).

Author	Ref	Type of Study	Pt	Radiotracer Scanner, Injected Activity and Delay before Acquisition	Comments
*Phosphaturic mesenchymal tumors (PMT)*					
John, J. (2019)	[38]	Retrospective	16	[^68^Ga]Ga-DOTA-TATE PET/CT(Siemens Biograph) 75–185 MBq, 30–45 min	Out of 16 patients with clinically suspected oncogenic osteomalacia 13 patients were found to be SSTR PET positive for a possible mesenchymal tumour. 10 patients underwent surgery, all of which biopsy confirmed PMT.
Paquet, M. (2018)	[40]	Prospective	15	[^68^Ga]Ga-DOTA-TOC PET/CT(Siemens Biograph mCT, Phillips GeminiTF16), 1.6 MBq/kg, 60 min.	9/15 identified suspect tumor, 8 removed surgically, all of them histologically proven to be PMT.
Singh, D. (2017)	[49]	Prospective	17	[^68^Ga]Ga-DOTA-NOC PET/CT (Siemens mCT biograph 64), 111–148 MBq, 45 ± 15 min.	DOTANOC PET/CT revealed 52 lesions in 17 patients with elevated FGF-23 and hypophosphatemia, where 11 were highly suspicious for culprit lesions. Subsequent anatomical imaging with CT/MRI showed concordant results in 7 out of 9 patients. These lesions were excised and histologically verified PMTs. Multiple lesions make it difficult to identify the culprit lesion.
Satyaraddi, A. (2017)	[41]	Retrospective	13	[^68^Ga]Ga-DOTA-TATE, [^18^F]FDG Scan details not given	DOTATATE revealed PMT in 9/9 patients, of whom 3 declined surgery. Other 6 had histologically verified PMTs.
González, G. (2017)	[42]	Retrospective	6	[^68^Ga]Ga-DOTA-TATE Scan details not given	Two tumors located using DOTATATE
El-Maouche, D. (2016)	[43]	Prospective	11	[^68^Ga]Ga-DOTA-TATE PET/CT(Siemens mCT), ca 185 MBq, 60 min [^111^In]In-OCT, [^18^F]FDG	6/11 patients had tumor successfully identified. DOTATATE identified 6/6, [^111^In]In-OCT and [^18^F]FDG both with 4/6.
Bhavani, N. (2016)	[44]	Retrospective	10	[^68^Ga]Ga-DOTA-NOC PET/CT (GE Discovery 609) 111–185 MBq, 60 min	DOTANOC detected PMT in 9/10 cases. 6/10 made full clinical recovery after complete resection.
Zhang, J. (2015)	[45]	Retrospective	54	[^68^Ga]Ga-DOTA-TATE PET/CT(Siemens Biograph 64) 111–148 MBq, ca 45 min	DOTATATE positive in 44 patients, where 33 had surgery to remove lesions. 32 histologically confirmed PMT. 10 who were not positive responded well to conservative treatment and thus PMT can be ruled out.
Agrawal, K. (2015)	[46]	Retrospective	6	[^68^Ga]Ga-DOTA-TATE PET/CT(GE Discovery STE-16) 1.5 MBq/kg, 45–60 min, [^18^F]FDG	[^18^F]FDG-PET identified PMT in 2/4 patients. DOTATATE identified PMT in 5/6 patients.
Jadhav, S. (2014)	[47]	Retrospective	16	[^68^Ga]Ga-DOTA-TATE PET/CT (Discovery STE) 74–111 MBq, 60–90 min. [^18^F]FDG, [^99^Tc]Tc-HYNIC-TOC	9/16 patients had tumor successfully identified. DOTATATE, HYNIC-TOC (tectrotyd) both identified 7/7, [^18^F]FDG 4/8.
Breer, S. (2014)	[14]	Prospective	5	[^68^Ga]Ga-DOTA-TATE PET/CT(Siemens Biograph), 58–110 MBq, 20 min, [^111^In]In-OCT	[^111^In]In-OCT SPECT-CT identified 1/5 tumors, DOTATATE-PET 5/5. Histologically confirmed PMT.
Clifton-Bligh, R. (2013)	[48]	Prospective	6	[^68^Ga]Ga-DOTA-TATE PET/CT, 103–226 MBq, 45–60 min	DOTATATE detected PMT in all 6 cases. 5/6 made full clinical recovery after resection. Patient with symptoms with residual on follow up PET.
*Juvenile angiofibroma*					
Gronkiewicz, Z. (2016)	[67]	Prospective	6	[^68^Ga]Ga-DOTA-TATE PET/CT (Siemens Biograph 64) 120–160 MBq, 60 min	DOTATATE showed uptake in areas matching the pathologic tissue in juvenile angiofibroma.
*Multiple myeloma*					
Sonmezoglu, K. (2017)	[71]	Prospective	21	[^68^Ga]Ga-DOTA-TATE PET/CT (Siemens Biograph 6 or GE Discovery V710), 100–150 MBq, 45–60 min. [^18^F]FDG	No significant difference was found between the two modalities in terms of lesion numbers detected in multiple myoma. However, diffuse bone marrow uptake of DOTATATE seems to be a predicting factor for overall survival.

## 4. Discussion

### 4.1. Inflammation

Activated macrophages were shown to express a large amount of SSTR2 making these a possible target for SSTR PET in inflammatory disease. Historically, inflammatory cells’ requirement for glucose has been the primary target for PET/CT with the glucose analog 2-deoxy-2-[^18^F]fluoro-D-glucose ([^18^F]FDG). This method has limitations in tissues with high glucose consumption such as the brain and heart, or in tissue with high background tracer uptake such as the kidneys. The papers in this section considered the utility of SSTR PET for investigating inflammation and in many cases compared the results with [^18^F]FDG PET.

#### 4.1.1. Diverse Inflammatory Processes

Two cases of Takayasu arteritis (TA) were reported by Tarkin et al. [15] where [^18^F]FDG PET proved useful in diagnosing TA but more limited in value for tracking therapeutic responses or detecting residual arteritis. SSTR PET/MRI accurately identified the lesions in both cases suggesting potential for further evaluation. Broadly speaking [^18^F]FDG is highly sensitive but relatively non-specific, and cannot accurately distinguish arteritis from metabolically active vascular remodeling [83]. SSTR tracers may provide an alternative.

Arora et al. [16] reported two cases of Graves orbitopathy where SSTR PET/CT identified lesions without physiological uptake in adjacent muscle tissue, suggesting higher sensitivity than [^18^F]FDG PET. Pichler et al. [17] showed similar findings in one patient.

Cheng et al. [18] reported a case where SSTR PET/CT incidentally revealed IgG4 mediated lymphadenopathy in a 60 year old male, confirmed on histology.

In an observational study of 14 patients with idiopathic pulmonary fibrosis Ambrosini et al. [20] reported SSTR tracer uptake directly corresponded to pathological areas on high-resolution CT (HRCT). A control group of nonspecific interstitial pneumonia showed faint tracer uptake and healthy individuals showed no uptake.

Fastrez et al. [19] conducted a pilot study of 12 patients with various degrees of endometriosis. SSTR PET/CT showed uptake in rectovaginal deep infiltrating endometriosis and adenomyoma, but not in superficial peritoneal endometriosis or ovarian endometrioma. The study concluded that SSTR PET cannot be used to diagnose endometriosis.

**Figure 3:**
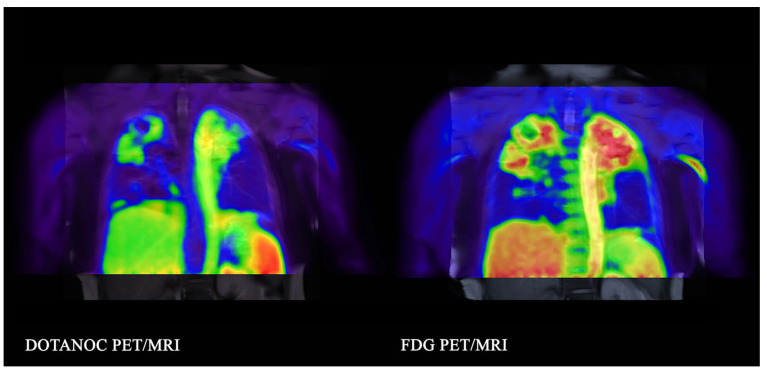
A 55 year old woman with pulmonary tuberculosis imaged with both DOTANOC and FDG PET on day 8 of treatment. Coronal images showing PET tracer uptake. DOTANOC uptake is more discrete. Reprinted unchanged with permission from ref. [21], Copyright 2020 Springer Nature.

SSTR tracer and [^18^F]FDG PET/MRI imaging were compared in 14 patients with pulmonary tuberculosis by Naftalin et al. [21]. [^68^Ga]Ga-DOTANOC was able to detect pulmonary lesions, but [^18^F]FDG was more sensitive for both active and subclinical lesions (see Figure 3).

In summary, these papers suggest that SSTR-PET tracers show uptake in diverse inflammatory processes, in some cases outperforming [^18^F]FDG PET regarding sensitivity and specificity. However, SSTR PET is generally less applicable than [^18^F]FDG, performing less well in tuberculosis and endometriosis. Tissue receptor diversity may underlie this variability.

#### 4.1.2. Sarcoidosis

Sarcoidosis is a heterogeneous, granulomatous disorder of unknown etiology. In the past, gallium-67 [^67^Ga]citrate scintigraphy was used for sarcoidosis but bears lower resolution/sensitivity and higher patient dose than PET and has been superseded by conventional radiology and [^18^F]FDG PET. Guidelines from European and American nuclear medicine and cardiac societies recommend the use of [^18^F]FDG PET in cardiac sarcoidosis diagnosis [84]. As few as 5% of patients with systemic sarcoidosis have clinical cardiac sarcoidosis, but autopsy studies reveal that as many as 70% have subclinical cardiac involvement. [^18^F]FDG PET otherwise plays a role in patients investigated for fever of unknown origin or lymphadenopathy where the eventual diagnosis is sarcoidosis.

In 11 studies with a total of 130 patients, SSTR PET was investigated as an alternative to [^18^F]FDG in systemic sarcoidosis. Sharma et al. [13] investigated 39 patients with sarcoidosis of whom 27 had symptomatic sarcoidosis with thoracic involvement, where [^68^Ga]Ga-DOTANOC uptake indicated increased disease activity in 25/27 patients (92%). In the asymptomatic group, 2/12 exhibited increased disease activity on [^68^Ga]Ga-DOTATOC PET. Nobashi et al. [22] compared [^68^Ga]Ga-DOTATOC PET to [^67^Ga] citrate scintigraphy (which is taken up in sarcoid lesions by mechanisms other than somatostatin receptors) in 20 patients. [^68^Ga]Ga-DOTATOC was positive in 19/20 patients vs. 17/20 in scintigraphy and demonstrated more lesions, suggesting SSTR PET is better at identifying sarcoid lesions; however, this can be explained by PET’s superior sensitivity compared to scintigraphy and not necessarily by higher lesion uptake.

**Figure 4:**
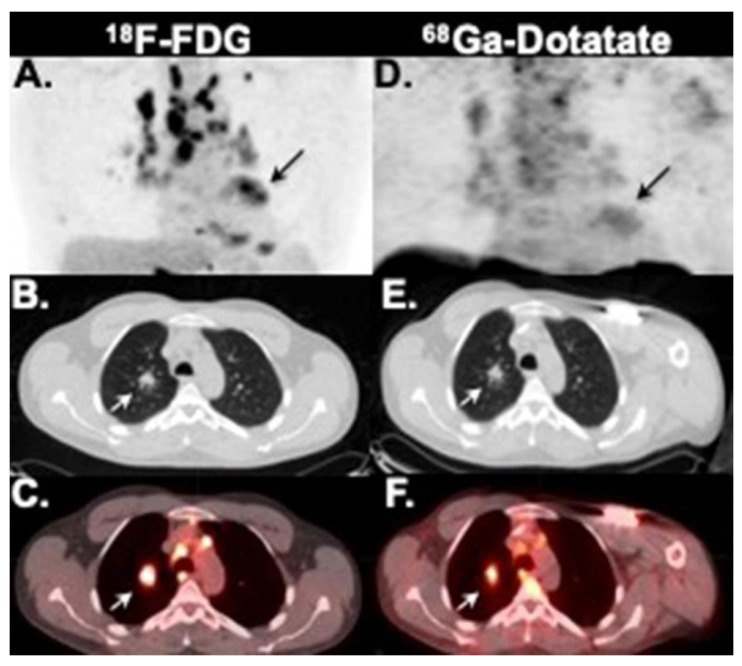
Patient with thoracic sarcoidosis. Both FDG and DOTATE PET images show extensive lymphadenopathy and cardiac inflammation (arrow on maximum intensity projections **A**,**D**) Axial CT (**B**,**E**) and fused PET-CT (**C**,**F**). Reprinted with permission from ref. [23] Copyright 2019 American Society of Nuclear Cardiology.

A total of eight studies consider SSTR tracers specifically for cardiac sarcoid. Bravo et al. [23] reported a pilot study with 13 patients and found SSTR PET less sensitive than [^18^F]FDG PET for detection of myocardial inflammation. Some representative results are shown in Figure 4.

In contrast Gormsen et al. [24] compared [^68^Ga]Ga-DOTANOC to [^18^F]FDG and showed a large proportion of [^18^F]FDG images were inconclusive whereas [^68^Ga]Ga-DOTANOC imaging illustrated excellent diagnostic accuracy, although with significant inter-observer variability. Pizarro et al. [25] reported 17 patients with cardiac sarcoid and varying degrees of immunosuppressant treatment imaged using SSTR PET and cardiac MR. They concluded SSTR PET may be more specific for acute inflammation and bear a potential role in treatment response. Other earlier or smaller studies demonstrated similar or improved performance of SSTR-PET tracers compared with [^18^F]FDG [26,27,28,29,30]. A case of neurosarcoidosis imaged with [^68^Ga]Ga-DOTATATE was reported by Unterrainer et al. [31].

These studies show variable comparative sensitivity of SSTR-PET tracers compared to [^18^F]FDG PET. One factor mentioned is the reliability of the [^18^F]FDG PET heart protocol that includes patients undertaking a low carbohydrate diet for 24–48 h, fasting for 12 h or more prior to imaging and a heparin bolus. This complicates the logistics of the procedure, adds the risk of poor compliance and may be a factor in differences in sensitivity of the studies. SSTR-PET tracers do not require a similar heart protocol.

#### 4.1.3. Other Causes of Myocardial Inflammation

Tarkin et al. [32] studied 12 patients with a history of myocardial infarction (MI) comparing [^68^Ga]Ga-DOTATATE to [^18^F]FDG PET within infarcted and non-infarcted segments. [^68^Ga]Ga-DOTATATE exhibited very low background myocardial binding; however, myocardial [^18^F]FDG was uninterpretable in five scans (42%) despite pre-scan fasting. [^68^Ga]Ga-DOTATATE imaging detected post infarction myocardial inflammation in both old and new ischemic injury. Close correlation between SSTR PET and MR in 12 patients with active peri/myocarditis or subacute myocardial infarction was reported by Lapa et al. [33]. Both studies suggest SSTR PET may have potential as a biomarker for cardiac remodeling.

#### 4.1.4. Atherosclerosis

Vulnerable plaques can be identified with SSTR-analogs by uptake in activated macrophages. There were three studies in this category. Lee, et al. [35] retrospectively investigated 50 patients originally screened with SSTR PET for NETs to observe whether there was incidental uptake in the thoracic aorta and found uptake correlating with cardiovascular risk factors, suggesting potential for imaging vulnerable plaque. Tarkin et al. [34] compared [^68^Ga]Ga-DOTATATE with [^18^F]FDG in coronary, carotid and aortic artery plaque in 42 patients. Both tracers distinguish culprit from non-culprit plaque in coronary and carotid arteries in patients with acute coronary syndrome (ACS), stroke, or transient ischemic attack (TIA); however, nearby background [^18^F]FDG uptake rendered coronary [^18^F]FDG scans uninterpretable in a significant number of patients (64%). [^68^Ga]Ga-DOTATATE scans were readable in all patients and supported the view that SSTR PET offers superior coronary imaging, excellent macrophage specificity and better power in discriminating high-risk versus low-risk coronary lesions. Both the study by Tarkin et al. [34] and a study by Pedersen et al. [36] use histopathological methods to correlate SSTR tracer uptake with SSTR2, CD (cluster of differentiation) 68 and CD163 expression in activated macrophages.

In summary, in coronary and vascular disease, SSTR PET shows some tracer uptake associated with active macrophages in inflammatory lesions, both acute and chronic, and contains a distinct advantage over [^18^F]FDG PET with much lower background uptake.

#### 4.1.5. Neuroinflammation

Dundar et al. [37] report three cases of incidentally detected stroke in oncology scans using [^68^Ga]Ga-DOTATATE, [^11^C]-choline and [^18^F]FDG tracers suggesting tracer uptake in neuroinflammation in regions of acute/subacute stroke.

### 4.2. Benign Neoplasia

Neuroendocrine tumors originate from a cell population normally expressing SSTRs and the well differentiated types of tumor typically overexpress these receptors. Several other neoplastic processes were found to exhibit increased expression of SSTRs as well. The reason is not fully understood, but is most likely linked to the role of somatostatin in regulating proliferation, mitogenesis and apoptosis [6]. A total of 46 studies were found investigating neoplastic processes other than NETs, consisting of a few exceptions with reports of incidental findings and small case studies.

#### 4.2.1. Tumor-Induced Osteomalacia and Phosphaturic Mesenchymal Tumors

Tumor-induced osteomalacia (TIO), also termed oncogenic osteomalacia, is a paraneoplastic syndrome typically caused by small benign mesenchymal tumors and characterized by hypophosphatemia (phosphaturic mesenchymal tumors, PMT). Tumor cells produce fibroblast growth factor 23 (FGF23) which in turn decreases proximal tubule reabsorption of phosphates and also inhibits vitamin D3 metabolism. This mobilizes calcium and phosphate from bones, and reduces osteoblastic activity which leads to bone tissue loss. It is diagnosed by identifying chronic hypophosphatemia, elevated FGF23 and decreased 1,25-OH2-Vitamin D [85]. Complete removal of the tumor is curative, which highlights the importance of locating the tumor. SSTRs were found on the cell membranes of active FGF23 secreting tumors with variable degrees of expression and thus SSTR analog tracers could be helpful in imaging [86]. A total of 30 studies involving 196 patients investigated TIO with SSTR PET. Single case studies comprised 16 of 30 reports, as can be expected with such a rare disease.

**Figure 5:**
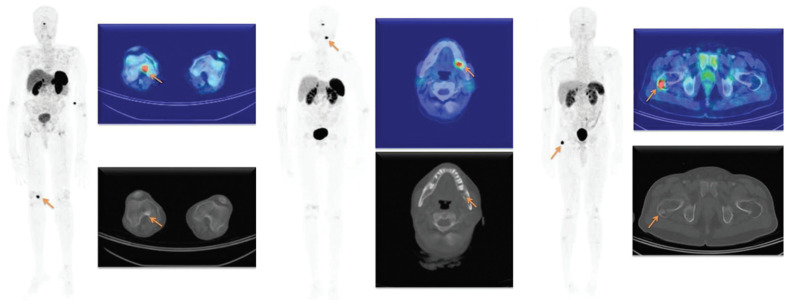
Example of 3 patients with tumour induced osteomalacia (TIO) where [^68^Ga]Ga-DOTATATE PET/CT localized culprit lesions. Lesions demonstrated intense tracer uptake on the whole body representative maximum intensity projection (MIP) and fused PET/CT images (indicated by arrows). Reprinted with permission from ref [38]. Copyright 2019 Indian Journal of Nuclear Medicine.

Zhang [45] retrospectively reviewed 54 patient histories with clinically suspected TIO. SSTR PET/CT was positive in 44 patients, among which 33 underwent surgery to remove lesions. Postsurgical pathological examination confirmed causative tumors in 32 patients whose symptoms diminished after surgery. El-Maouche et al. [43] performed a prospective study of 11 patients comparing [^68^Ga]Ga-DOTATATE with [^18^F]FDG and [^111^In]In-pentetreotide (octreoscan). Six patients had a culprit tumor successfully identified. [^68^Ga]Ga-DOTATATE detected 6/6, [^18^F]FDG and octreoscan both found 4/6 suggesting [^68^Ga]Ga-DOTATATE exhibited better sensitivity than the alternatives. Small studies by Agrawal et al. [46] and Jadhav et al. [47] also reported higher sensitivity and specificity for SSTR tracers compared with [^18^F]FDG. Multiple other case and small studies summarized in Table 2 report localization of culprit lesions in patients with TIO using SSTR tracers [14,38,39,40,41,42,44,48,49,50,51,52,53,54,55,56,57,58,59,60,61,62,63,64,65,66]. An example showing culprit lesions identified in 3 patients using [^68^Ga]Ga-DOTATATE PET-CT from a small study by John et al. [38] is shown in Figure 5.

In summary, PMT is a rare lesion that can arise in the head and neck or lower extremities. PET is typically performed as a whole body protocol (+/−extremities) and therefore lends itself to uncovering primary lesions of unknown origin. The data suggest that SSTR tracers outperform [^18^F]FDG and is slowly becoming accepted as a sensitive imaging method for localizing the culprit lesion [87].

#### 4.2.2. Other Benign Tumors

Juvenile angiofibroma presents with rapidly growing vessels, associated with a higher SSTR2A expression in patients. Gronkiewicz et al. [67] performed a prospective study in six patients using SSTR PET that showed weak uptake in areas matching the pathologic tissue. In all cases, immunohistochemical examination revealed SSTR2A with a high staining index. The weak uptake was suggested to be due to intracellular localization of receptors.

A case of an incidental enchondroma in the right tibia was reported by Mahajan et al. [68] imaged on SSTR PET suggesting overexpression of SSTR in enchondromas.

Vertenten et al. [69] described a case of a patient with known pancreatic cancer showing [^68^Ga]Ga-DOTATATE uptake in the spine suspected to be metastases. CT showed characteristic appearances of multiple hemangiomas. 

Liu et al. [70] described a case where [^68^Ga]Ga-DOTATATE uptake was observed in an incidentally detected uterine leiomyoma.

These benign incidental findings with SSTR tracer uptake highlights the potential for false positive findings on SSTR-PET scans performed for NETs.

### 4.3. Malignant Disease

#### 4.3.1. Multiple Myeloma (MM) 

Multiple myeloma (MM) is a neoplasm of plasma B cells characterized by bone marrow infiltration with an overproduction of a monoclonal plasma cell population and antibodies, often leading to skeletal lesions, hypercalcemia and kidney dysfunction [88]. Sharma et al. [72] described a patient with MM with two osteolytic lesions demonstrating high tracer uptake on SSTR PET/CT. Sonmezoglu et al. [71] reported a prospective study of 21 patients with known MM where all patients were scanned with both [^18^F]FDG PET/CT and [^68^Ga]Ga-DOTATATE PET/CT. No significant difference was found between the two modalities in terms of lesion numbers detected. However, diffuse bone marrow uptake of [^68^Ga]Ga-DOTATATE seemed to be a predictive factor for reduced overall survival. Some lesions visible on [^18^F]FDG were not detected on SSTR PET and the authors hypothesized that this might be influenced by a mismatch between receptor expression and tracer-binding affinity, particularly for SSTR3 and 5 receptors.

#### 4.3.2. Epithelioid Hemangioendothelioma (EHE)

Epithelioid hemangioendothelioma (EHE) is a rare low-grade tumor originating from vascular endothelial or pre-endothelial cells with the potential to metastasize and recur [89]. Derlin et al. [74] described a case where [^68^Ga]Ga-DOTATATE and [^18^F]FDG identified vertebral metastasis from EHE. [^68^Ga]Ga-DOTATATE demonstrated a higher tumor to background ratio. Long et al. [73] reported a case of EGE in the right upper mediastinum located with [^68^Ga]Ga-DOTATATE, pathologically confirmed after resection. This case was also associated with TIO (see Section 4.2.1). A recent consensus statement on EHE diagnosis and treatment [89] mentions [^18^F]FDG PET as a useful adjunct. However, it remains yet to be established whether SSTR PET represents a better option.

#### 4.3.3. Renal Cell Carcinoma Metastases

Vamadevan et al. [75] reported a case where metastasis from clear cell renal cell carcinoma (ccRCC) in the pancreas was incidentally detected on SSTR PET/CT. Vamedevan et al. [76] subsequently continued to report progression and a metastasis in the patient’s left thigh. Höög et al. [77] retrospectively compared [^68^Ga]Ga-DOTATOC in one patient vs. [^18^F]FDG in another patient, both diagnosed with ccRCC and with lesions suspicious for metastases. [^18^F]FDG showed no uptake in an adrenal metastasis. [^68^Ga]Ga-DOTATOC identified a pancreatic metastasis. Both lesions were histopathologically proven to be of ccRCC origin and to demonstrate SSTR2 expression. These case reports highlight both the potential utility of SSTR tracers for RCC metastases but also the need to consider differential diagnoses on SSTR scans performed for neuroendocrine tumors.

#### 4.3.4. Various Malignancies Reported as Case Reports

Braat et al. [78] reported of a case where SSTR PET detected a gastrointestinal stromal tumor (GIST) in the appendix. Savelli et al. [82] reported a case where SSTR PET detected relapse of a previously resected oligodendroglioma. The article suggested the consideration of further research in theragnostics based on the significant tracer uptake and contrast and potential for peptide receptor radionuclide therapy (PRRT). Freesmeyer et al. [79] reported a case where SSTR PET incidentally detected a hepatocellular carcinoma (HCC) with skeletal metastasis. Shamim et al. [80] reported a case where SSTR PET incidentally detected metastasis from choroidal melanoma in the pancreas. Qin et al. [81] compared [^68^Ga]Ga-DOTATATE and [^18^F]FDG uptake in osseous lesions from synchronous multifocal osteosarcoma. [^18^F]FDG did not identify lesions; however, [^68^Ga]Ga-DOTATATE accumulated in lesions which were later resected and pathologically confirmed.

In summary, these studies and cases highlight that SSTR tracers provide a potential alternative to [^18^F]FDG for imaging certain malignancies other than NET. In NET tumors, [^18^F]FDG and SSTR tracers are often complementary in their avidity for tumor types, reflecting the degree of differentiation and proliferation and this observation appears to apply to other tumor types. [^18^F]FDG however, is a well-established and accessible technique with relatively well understood kinetics and metabolism whereas SSTR tracers can bear potentially great specificity but are dependent on SSTR expression, which is often much less well understood in many lesion types.

## 5. Conclusions

SSTR tracers bear applications beyond its classical use in neuroendocrine tumors and meningiomas. Numerous tissues express SSTR1-5 and pathological processes originating from these tissues can provide targets for SSTR imaging. Use of SSTR PET for investigating tumor-induced osteomalacia and localization of culprit lesions is virtually an established technique.

Inflammation, particularly associated with sarcoidosis and atherosclerosis, represents another application with promising results. Activated macrophages express SSTR and take up SSTR tracers which may provide a level of specificity not available with [^18^F]FDG PET. In addition, SSTR tracers exhibit advantageously low background uptake in the heart, brain and arterial wall compared with [^18^F]FDG.

Other applications remain a work in progress, where there exist many case reports but a relative dearth of cohort studies. [^18^F]FDG PET provides the established comparative PET method in many cases, and frequently shows different sensitivity reflecting the different underlying physiology and uptake mechanism. In analogy to NET, SSTR PET can be performed as a complementary PET technique to [^18^F]FDG PET, particularly where the lesion is likely to be well differentiated and/or express SSTR receptors.

Moreover, new applications for SSTR tracers bear the advantage of potentially novel theragnostic opportunities for treatment with SSTR-based peptide receptor radionuclide therapy (PRRT).

## Figures and Tables

**Table 1:** Distribution of somatostatin receptors in normal human tissue (adapted from [3,4,5,6,7]).

Tissue	Localization	Receptor Subtype
*Lymphatic:*		SST2 (probable)
Lymph nodes	Germinal centers	
Peyers Patches	Germinal centers	
Solitary follicles (colon)	Germinal centers	
Appendix	Germinal centers	
Spleen	Red pulp	SST2-3
Thymus	Medulla	SST1-3
*Immune cells:*		
Macrophages		SST2
Dendritic cells		SST2
B lymphocytes		SST3
T lymphocytes		SST1-5
*Kidneys:*		SST2 (probable)
Cortex	Proximal tubules	
Medulla	Vasa recta	
Prostate	Smooth muscle	SST2
Thyroid gland	Epithelial cells	SST2
*GI-tract:*		
Stomach		SST1-4
Small intestine		SST1, SST5
Liver		SST3
Pancreas	β cells	SST2
	α cells	SST3
	δ cells	SST5
Adrenal glands		SST2
Lung		SST4
Heart		SST4
